# The Fungal Effector Avr-Pita Suppresses Innate Immunity by Increasing COX Activity in Rice Mitochondria

**DOI:** 10.1186/s12284-021-00453-4

**Published:** 2021-01-14

**Authors:** Jingluan Han, Xiaoyu Wang, Fengpin Wang, Zhe Zhao, Gousi Li, Xiaoyuan Zhu, Jing Su, Letian Chen

**Affiliations:** 1 State Key Laboratory for Conservation and Utilization of Subtropical Agro-Bioresources, South China Agricultural University, Guangzhou, 510642 China; 2 Guangdong Provincial Key Laboratory of Protein Function and Regulation in Agricultural Organisms, South China Agricultural University, Guangzhou, 510642 China; 3 College of Life Sciences, South China Agricultural University, Guangzhou, 510642 China; 4 Guangdong Provincial Key Laboratory of High Technology for Plant Protection, Plant Protection Research Institute, Guangdong Academy of Agricultural Sciences, Guangzhou, 510640 China

**Keywords:** Innate immunity, Effector, Avr-Pita, Mitochondrion, Cytochrome *c* oxidase (COX), Reactive oxygen species (ROS)

## Abstract

**Background:**

Avr-Pita was the first effector identified in the blast fungus (*Magnaporthe oryzae*)–rice (*Oryza sativa*) pathosystem. However, the molecular mechanism underlying its effects on the host plant has remained a long-standing mystery.

**Results:**

Here, we report that ectopically expressing *Avr-Pita* in rice enhances susceptibility to *M. oryzae* and suppresses pathogen-associated molecular pattern (PAMP)-triggered defense responses. Avr-Pita targets the host mitochondria and interacts with the cytochrome *c* oxidase (COX) assembly protein OsCOX11, a key regulator of mitochondrial reactive oxygen species (ROS) metabolism in rice. Overexpressing *Avr-Pita* or *OsCOX11* increased COX activity and decreased ROS accumulation triggered by the fungal PAMP chitin. *OsCOX11*-overexpressing plants showed increased susceptibility to *M. oryzae*, whereas *OsCOX11*-knockdown plants showed resistance to *M. oryzae*.

**Conclusions:**

Taken together, these findings suggest that the fungal pathogen *M. oryzae* delivers the effector Avr-Pita to the host plant, where it enhances COX activity thus decreasing ROS accumulation. Therefore, this effector suppresses host innate immunity by perturbing ROS metabolism in the mitochondria.

**Supplementary Information:**

The online version contains supplementary material available at 10.1186/s12284-021-00453-4.

## Introduction

Plants have evolved sophisticated mechanisms to perceive and respond to pathogen attack, such as pathogen-associated molecular pattern (PAMP)-triggered immunity (PTI) and effector-triggered immunity (ETI) (Liu et al., 2014). PTI and ETI are associated with immune responses including the rapid influx of calcium, a burst of reactive oxygen species (ROS), mitogen activated protein kinase (MAPK) phosphorylation cascades, callose deposition, pathogenesis-related (*PR*) gene expression, and the biosynthesis of antimicrobial compounds (Bigeard et al., 2015).

Rice blast caused by the hemibiotrophic fungal pathogen *Magnaporthe oryzae* (*M. oryzae*) is a devastating disease that threatens global food security (Dean et al., 2012; Skamnioti & Gurr, 2009). The rice–*M. oryzae* pathosystem is a well-known model for studying plant–pathogen interactions (Wilson & Talbot, 2009). Over the past decades, several *M. oryzae* avirulence (Avr) effector genes have been cloned and characterized, including *PWL1*, *PWL2*, *PWL3*, *PWL4*, *Avr-Pita*, *ACE1*, *AvrPiz-t*, *Avr-Pia*, *Avr-Pii*, *Avr-Pik*/*km*/*kp*, *Avr1-CO39*, *Avr-Pi9*, and *Avr-Pib (Zhang & Xu,* 2014*;* *Zhang et al.,* 2018*)*. However, the virulence functions of only a few Avr proteins have been elucidated. AvrPiz-t manipulates host immunity by interacting with the RING-type ubiquitin E3 ligases APIP6 and APIP10, thereby inhibiting their E3 ligase activity and promoting their degradation, whereas APIP6 and APIP10 degrade AvrPiz-t (Park et al., 2012; Park et al., 2016). Inside the host cell, AvrPiz-t targets the bZIP-type transcription factor APIP5 to eliminate APIP5-triggered cell death and promote tissue necrosis (Wang et al., 2016). AvrPiz-t also interacts with the potassium channel OsAKT1 by competing with the cytosolic protein kinase OsCIPK23 to modulate K^+^ channel activity (Shi et al., 2018). Moreover, AvrPiz-t interacts with the nucleoporin protein APIP12 to suppress host immunity (Tang et al., 2017). Another well-documented Avr protein, AvrPii, disrupts host immune systems by interacting with NADP-MALIC ENZYME 2 (OsNADP-ME2) to inhibit NADP-ME activity or by interacting with two exocytosis complex OsExo70 proteins (Fujisaki et al., 2015; Singh et al., 2016). These evidences indicate that Avr proteins may target to various host factors to manipulate host immunity. However, the function of Avr protein is still largely unknown.

Although Avr-Pita was the first fungal effector isolated from the *M. oryzae*–rice pathosystem (Jia et al., 2000; Orbach et al., 2000), our mechanistic understanding of its role in this pathosystem remains limited. Avr-Pita is a putative neutral zinc metalloprotease with a signal peptide at its N-terminus that is thought to be recognized by the nucleotide binding site-leucine rich repeat domain (NBS-LRR) of protein Pi-ta, which triggers host immune responses in rice cells (Bryan et al., 2000; Orbach et al., 2000). During the infection processes, Avr-Pita accumulates in the biotrophic interfacial complex (BIC) structure and is delivered into host cells *via* invasive *M. oryzae* hyphae (Khang et al., 2010). However, where and how Avr-Pita functions inside host cells remains unclear.

ROS play key roles in plant defense signaling. In response to the perception of pathogen invasion, plants activate a rapid ROS burst leading to the hypersensitive response (HR), programmed cell death (PCD), and kinase cascade (Smirnoff & Arnaud, 2019). High concentrations of ROS also function as toxicants to the pathogen (Kou et al., 2019). ROS are produced in several cellular compartments including the plasma membrane, mitochondria, peroxisomes, and chloroplasts (Qi et al., 2018; Smirnoff & Arnaud, 2019). The generation of ROS by the plasma membrane enzyme NADPH oxidase during plant defense responses has been well documented. The mitochondrion is a major organelle that produces ROS *via* Complexes I, II, and III of the mitochondrial electron transport chain (METC) (Vanlerberghe, 2013). However, little is known about the association of mitochondrial METC Complexes I and III with defense responses (Vidal et al., 2007).

Here, we demonstrate that Avr-Pita targets host mitochondria and interacts with cytochrome *c* oxidase (COX) assembly protein OsCOX11 of the METC, which promotes the activity of Complex IV of the METC, thereby inhibiting ROS accumulation and suppressing innate immunity in rice.

## Results

### The Avr-Pita Effector Suppresses Defense Responses in Rice

To investigate the function of Avr-Pita inside rice cells, we expressed Avr-Pita in rice cells. To this end, we generated rice suspension cultures harboring the estradiol-induced construct *P*_*XVE*_*::Avr-Pita* (Fig. S1a). Estradiol treatment significantly induced the expression of *Avr-Pita* in these lines (Fig. S1c and S1e)*. T*he expression of the early defense response gene *PHENYLALANINE AMMONIA LYASE 1* (*OsPAL1*) was markedly suppressed in response to estradiol treatment in these lines at the indicated time points (Fig. S1f), suggesting that inducing the expression of *Avr-Pita* inhibits basic defense responses in rice cells.

**Fig. 1:**
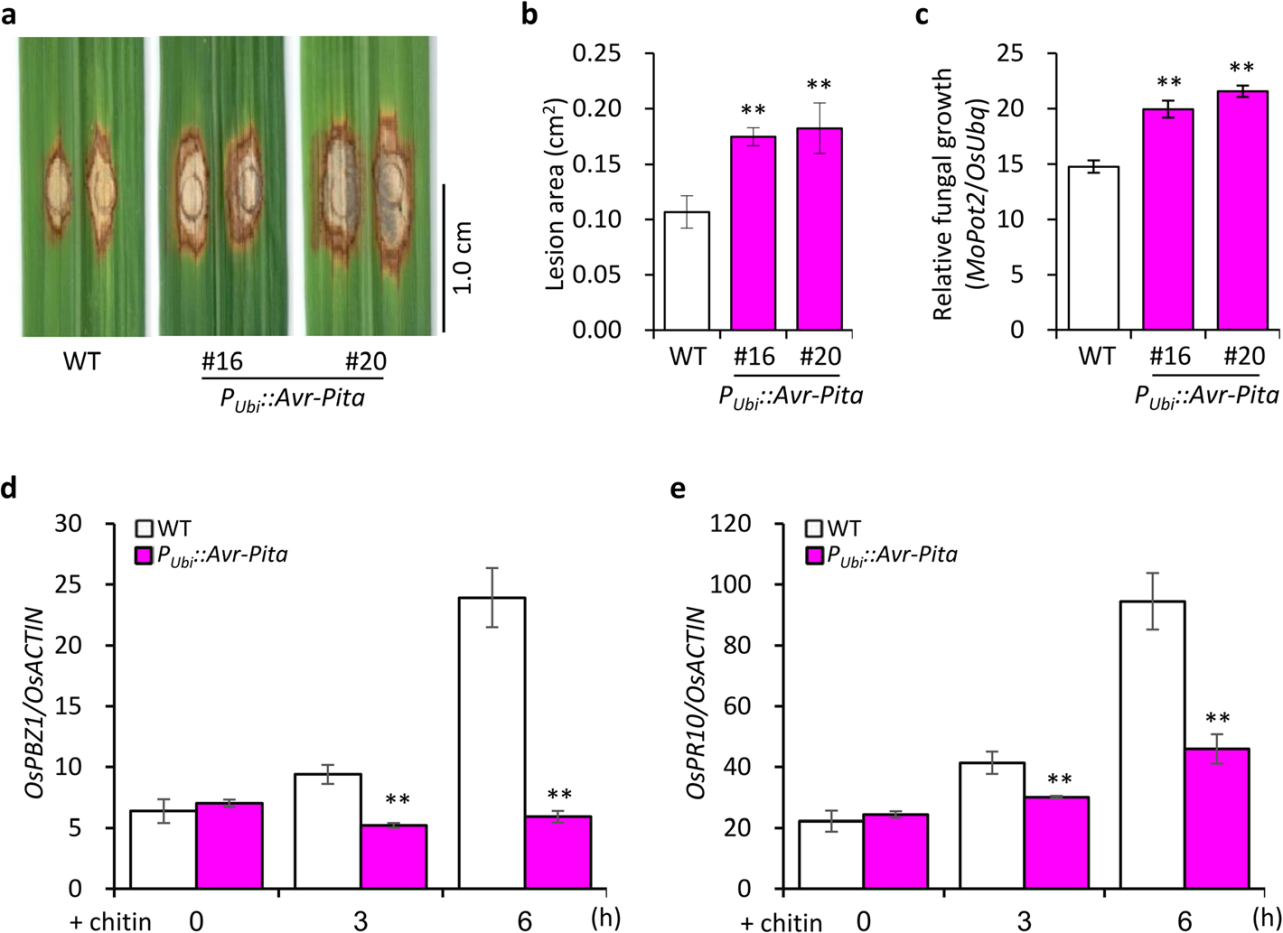
Overexpressing *Avr-Pita* suppresses basal resistance to *M. oryzae* in rice. **a** Disease symptoms of *P*_*Ubi*_*::Avr-Pita* lines and wild-type (WT) plants at 12 days post inoculation (dpi) with compatible isolate 10–441*.* **b** Lesion area was photographed and measured using ImageJ software. Data are shown as mean ± standard error (SD) (**P < 0.01, *n* > 12). **c** Relative fungal biomass of *M. oryzae* in inoculated leaves, as quantified by qPCR comparing the DNA amounts of the fungal gene *MoPot2* and the rice gene *OsUbq*. Data are shown as mean ± SD, (**P < 0.01, *n* = 3). **d** Expression of Os*PBZ1* in the leaves of *P*_*Ubi*_*::Avr-Pita* lines following chitin treatment. **e** Expression of Os*PR10* in *P*_*Ubi*_*::Avr-Pita* lines following chitin treatment. Data were normalized to the expression level of *OsACTIN*. Data are shown as mean ± SD (**P < 0.01, *n* = 3)

We also generated stably transformed *P*_*Ubi*_*::Avr-Pita* rice plants overexpressing *Avr-Pita* under the control of the maize (*Zea mays*) *Ubiquitin* promoter (Fig. S1a). Although the overexpression of *Avr-Pita* in the leaves of *P*_*Ubi*_*::Avr-Pita* plants was confirmed by quantitative reverse-transcription PCR (qRT-PCR) (Fig. S1d and S1g), ectopic overexpression of *Avr-Pita* did not significantly affect the morphology of rice plants in terms of growth and development (Fig. S1b). To test pathogen resistance, we inoculated the *P*_*Ubi*_*::Avr-Pita* lines with *M. oryzae* isolate 10–441 (without *Avr-Pita*) by punch inoculation. The *P*_*Ubi*_*::Avr-Pita* plants had larger disease lesions and higher relative fungal biomass than wild-type (WT) plants at 12 days post inoculation (dpi) (Fig. 1a–c). These results indicate that ectopic expression of *Avr-Pita* enhances rice susceptibility to *M. oryzae*.

To further investigate the PAMP-triggered defense response in rice, we treated the *P*_*Ubi*_*::Avr-Pita* lines with chitin and monitored the expression of *PR* genes. The expression of the *PR* genes *PROBENAZOLE-INDUCIBLE 1* (*OsPBZ1*) and *OsPR10* was significantly suppressed in *P*_*Ubi*_*::Avr-Pita* *vs.* WT plants, as indicated by qRT-PCR (Fig. 1d and e), indicating that Avr-Pita suppresses chitin-triggered PTI in rice.

### Avr-Pita Interacts with OsCOX11 in Rice Mitochondria

**Fig. 2:**
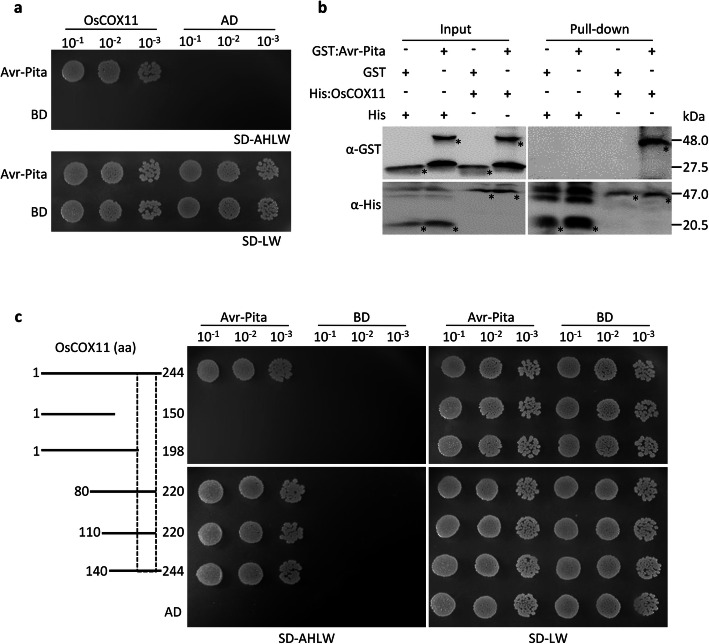
Avr-Pita interacts with OsCOX11. **a** Analysis of protein–protein interactions between BD:Avr-Pita (mature protease) and AD:OsCOX11 *via* yeast-two-hybrid (Y2H) assay. Yeast cells harboring the indicated bait and prey plasmids were diluted 10×, 100×, or 1000× and spotted onto selective media SD-LW and SD-AHLW. **b** In vitro pull-down assays were conducted with fusion proteins (GST:Avr-Pita and His:OsCOX11) using His-Ni beads and detected by immunoblot analysis with anti-GST and anti-His antibodies. Asterisks indicate the target protein. **(c)** Deletion assays of OsCOX11 to test interactions with Avr-Pita. A series of truncated OsCOX11 proteins were tested for interaction with Avr-Pita *via* Y2H

To explore how Avr-Pita suppresses defense responses in plants, we conducted yeast two-hybrid (Y2H) assays using Avr-Pita^49–224^ (processed mature protease) as bait and identified six candidate proteins (Table S1). We selected the mitochondrial COX assembly protein, OsCOX11, for further study. The interaction between Avr-Pita and OsCOX11 was further validated using full-length Avr-Pita protein in the Y2H system (Fig. 2a). We confirmed the interaction of OsCOX11 and Avr-Pita *via* a pull-down assay using His:OsCOX11 and GST:Avr-Pita (Fig. 2b).

To narrow down the specific region of OsCOX11 that physically interacts with Avr-Pita, we generated a series of truncated OsCOX11 fragments for Y2H assays. The OsCOX11^140–220^ fragment was identified as a sufficient region responsible for the interaction with Avr-Pita, while OsCOX11^199–220^ fragment (containing the 5th β sheet region) was a critical region responsible for the interaction (Fig. 2c). However, no orthologs of OsCOX11, such as AtCOX11 (*Arabidopsis thaliana, A. thaliana*)*,* ScCOX11 (*Saccharomyces cerevisiae, S. cerevisiae*), and MoCOX11 (*M. oryzae*), were able to interact with Avr-Pita (Fig. S2a and S2b). These results suggest that fungal Avr-Pita specifically binds to rice OsCOX11 *via* the specific region of OsCOX11.

**Fig. 3:**
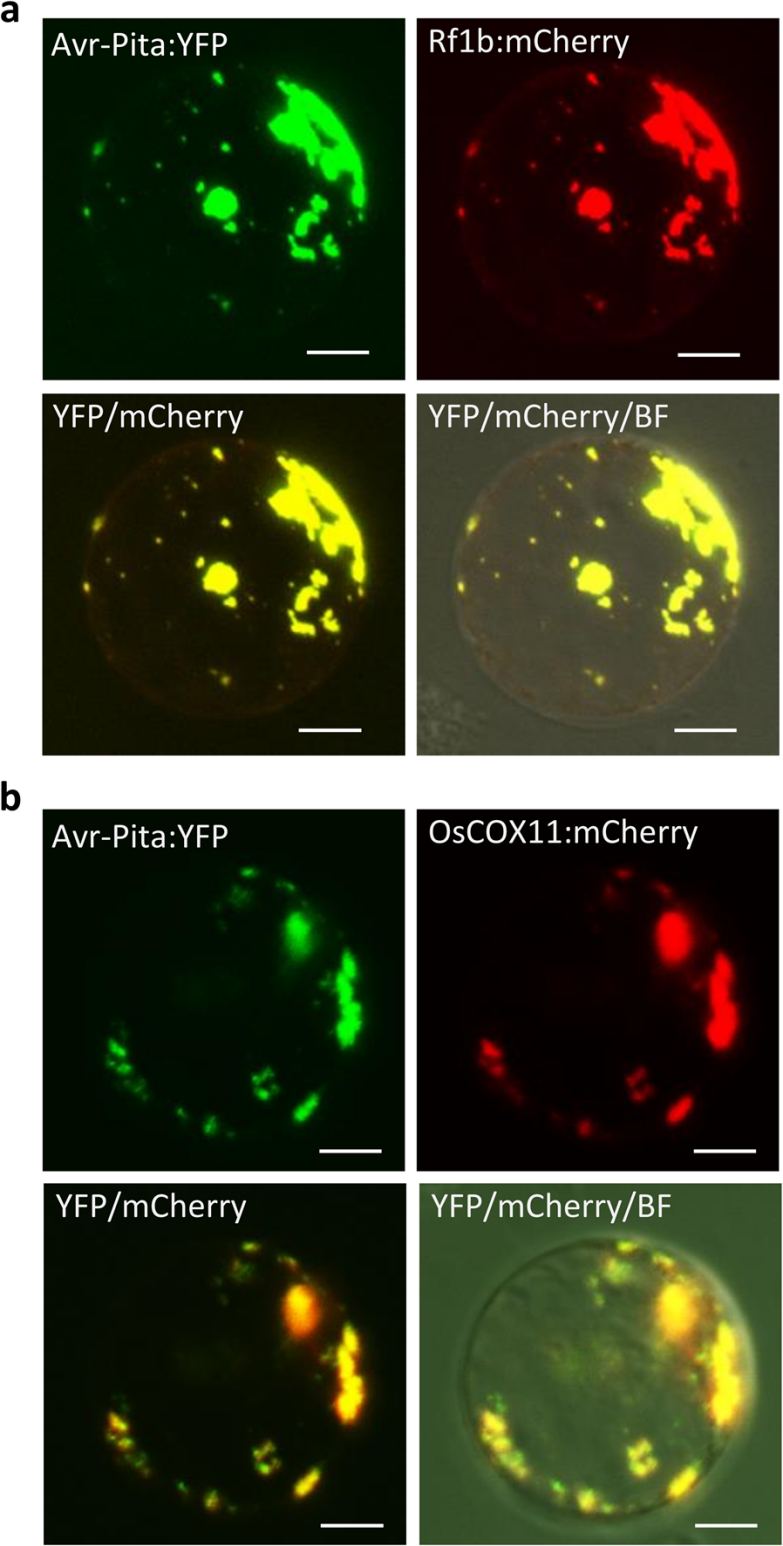
Avr-Pita and OsCOX11 co-localize to rice mitochondria. **a** *Avr-Pita:YFP* and mitochondrial marker *Rf1b:mCherry* were co-expressed in rice protoplasts. YFP and mCherry fluorescent signals overlapped in the mitochondria. **b** *Avr-Pita:YFP* and *OsCOX11:mCherry* were co-expressed in rice protoplasts. YFP and mCherry fluorescent signals overlapped in the mitochondria. Scale bars = 10 μm

To explore the subcellular localization of Avr-Pita inside plant cells, we generated rice protoplasts and onion epidermal cells transiently expressing Avr-Pita:yellow fluorescent protein (YFP) and examined them by confocal microscopy. Avr-Pita:YFP perfectly co-localized with the mitochondrial marker Rflb:mCherry (Wang et al., 2006) in rice protoplasts and with the mitochondrial dye MitoTracker in onion epidermal cells (Fig. 3a and S3a). Avr-Pita:YFP also co-localized with OsCOX11:mCherry in mitochondria (Fig. 3b and S3b). These results indicate that the fungal effector Avr-Pita targets the host mitochondria and binds OsCOX11, a subunit of the COX complex.

### OsCOX11 Negatively Regulates Disease Resistance

**Fig. 4:**
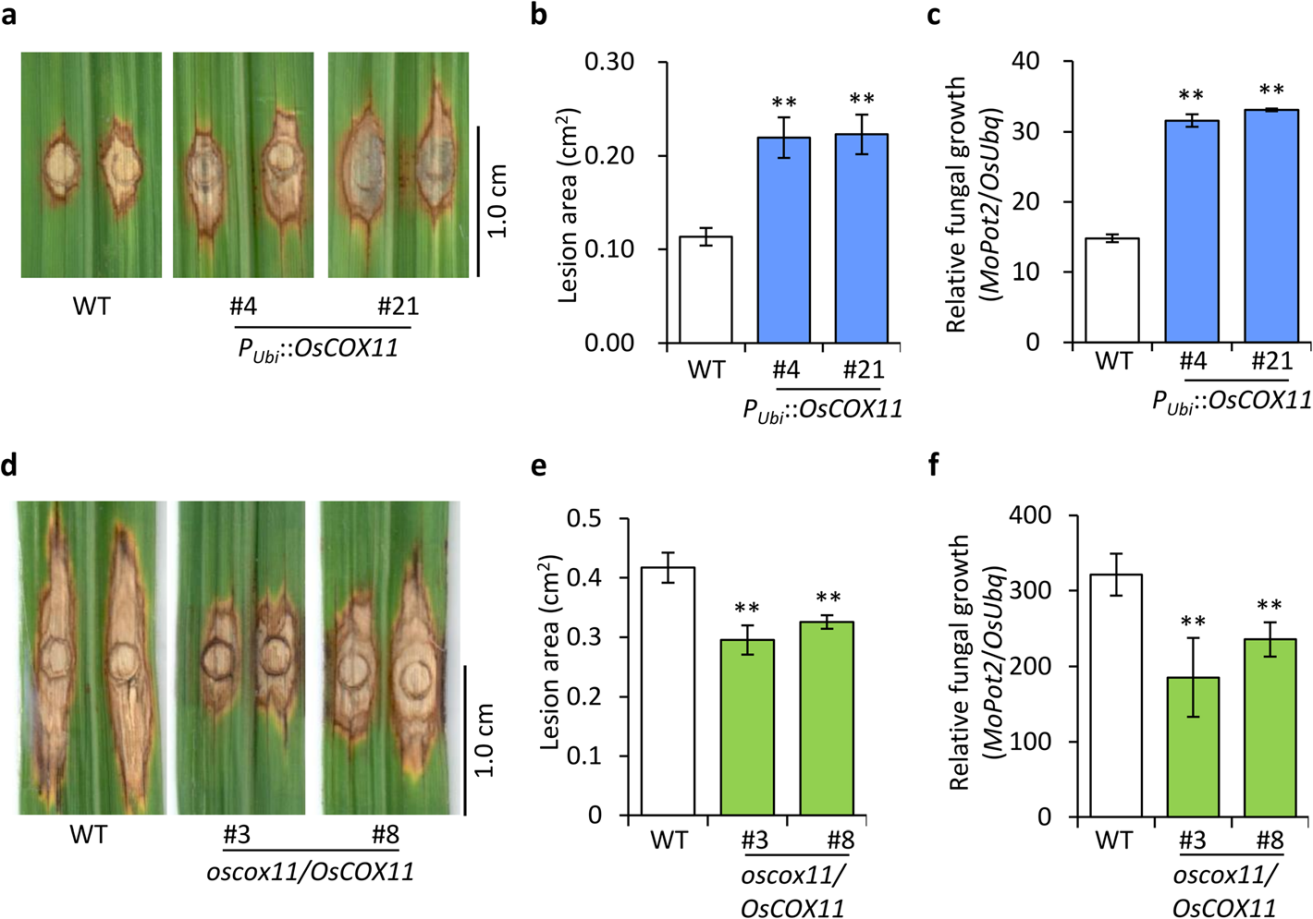
OsCOX11 functions as a negative regulator of basal resistance against *M. oryzae.* **a** Disease symptoms of *P*_*Ubi*_*::OsCOX11* and WT plants in response to *M. oryzae* isolate 10–441. The inoculated leaves were photographed at 12 dpi. **b** Lesion area was measured with ImageJ software. Data are shown as the mean ± SD (**P < 0.01, *n* > 12). **c** Fungal biomass in inoculated leaves, as quantified by qPCR comparing the DNA amounts of the fungal gene *MoPot2* and the rice gene *OsUbq*. Data are shown as mean ± SD (**P < 0.01, *n* > 12). **d** Disease symptoms of *oscox11/OsCOX11* and WT plants inoculated with *M. oryzae* isolate 13–219. The leaves were photographed at 12 dpi. **e** Lesion area was measured with ImageJ software. Data are shown as mean ± standard error (SD) (**P < 0.01, *n* > 12). **f** Relative fungal biomass on *M. oryzae*-inoculated leaves, as measured by qPCR. Data are shown as mean ± SD (**P < 0.01, *n* > 12)

To investigate the role of OsCOX11 in plant resistance to *M. oryzae*, we generated *OsCOX11-*overexpressing transgenic lines (harboring the *P*_*Ubi*_*::OsCOX11* construct) for functional studies (Fig. S4a). When the *P*_*Ubi*_*::OsCOX11* plants were inoculated with *M. oryzae* strain 10–441, they developed larger disease lesions (Fig. 4a and b) and more fungal biomass (Fig. 4c) than the control at 12 dpi.

To determine whether OsCOX11 plays a role in disease resistance, we produced *OsCOX11*-silenced and -knockout plants by RNAi and CRISPR/Cas9-mediated gene editing, respectively. Surprisingly, we failed to obtain homozygous knockout mutants (data not shown), indicating that OsCOX11 plays a crucial role in plant growth and development and that the homozygous mutant may be lethal. Therefore, we used the heterozygous *OsCOX11* knockout mutant (*oscox11*/*OsCOX11*) for functional analysis (Fig. S4b and S4c) of disease resistance. Following inoculation, the disease lesions of *oscox11*/*OsCOX11* plants were smaller than those of WT plants (Fig. 4d and e), indicating that *oscox11*/*OsCOX11* plants are resistant to *M. oryzae*. Consistent with this, the *OsCOX11*-RNAi lines were also more resistant to *M. oryzae* than WT plants (Fig. S4d, S4e and S4f). Moreover, the fungal biomass was significantly reduced in the *oscox11*/*OsCOX11* and *OsCOX11*-RNAi lines (Fig. 4f and S4g), indicating that OsCOX11 is a negative regulator of plant resistance to *M. oryzae*.

### Avr-Pita Hampers Plant Resistance by Promoting the COX Activity in ROS Metabolism

**Fig. 5:**
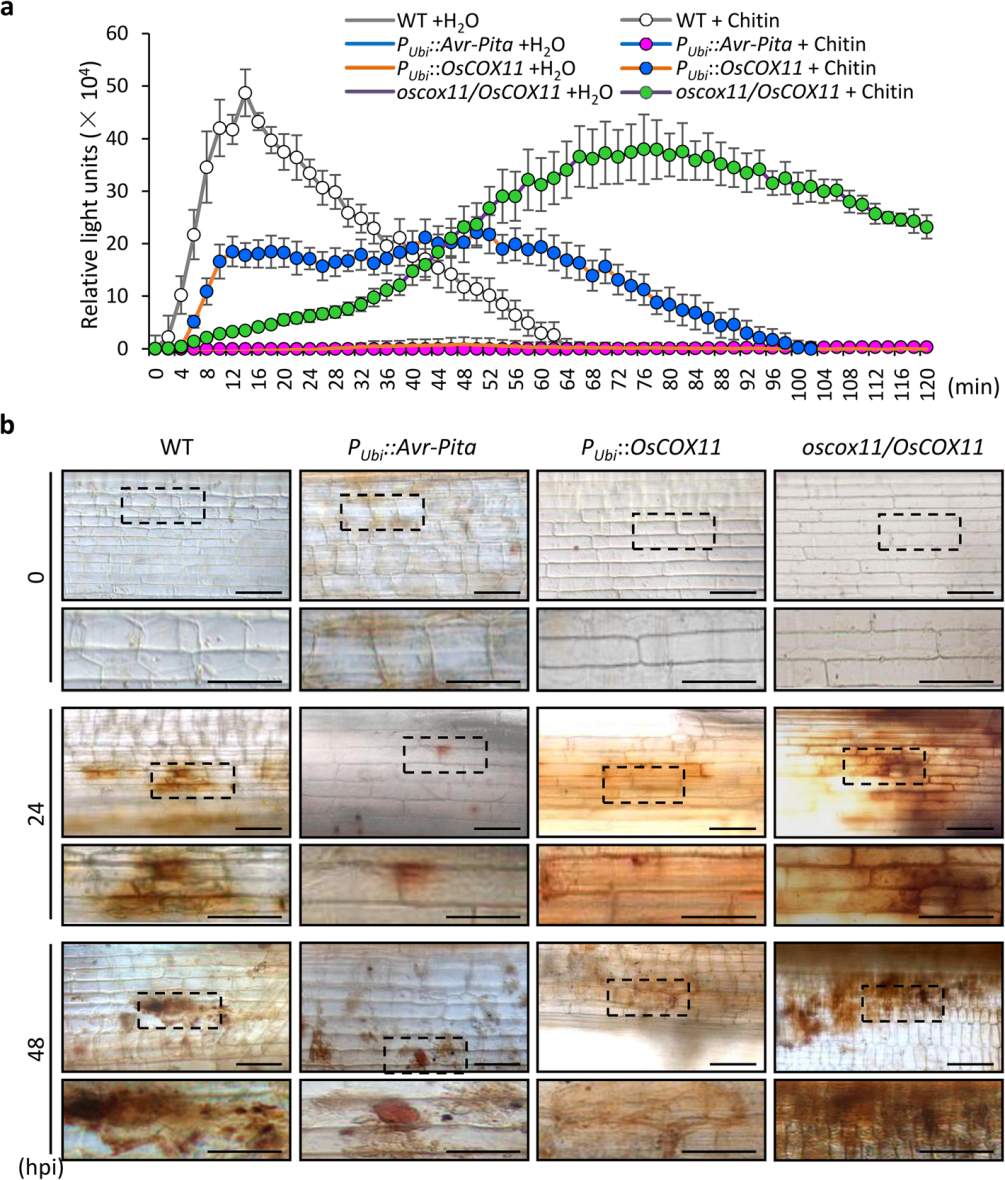
Overexpressing *Avr-Pita* or *OsCOX11* suppresses chitin-triggered ROS accumulation in rice. **a** Chitin-induced ROS accumulation in WT, *P*_*Ubi*_::*Avr-Pita*, *P*_*Ubi*_*::OsCOX11*, and *oscox11/OsCOX11* cells, as measured using a luminol-chemiluminescence assay. Data are shown as mean ± SD (**P < 0.01, *n* = 4). **b** ROS accumulation detected with DAB staining in WT, *P*_*Ubi*_*::Avr-Pita*, *P*_*Ubi*_*::OsCOX11*, and *oscox11/OsCOX11* plants after inoculation with *M. oryzae*. The images were taken at 0, 24, and 48 h post inoculation (hpi). Scale bars = 50 μm

To understand the effect of the relationship between Avr-Pita and OsCOX11 on plant resistance, we examined chitin-induced ROS accumulation in various transgenic plants using a luminol-based chemiluminescence assay. ROS accumulation was significantly suppressed in *P*_*Ubi*_*::Avr-Pita* and *P*_*Ubi*_*::OsCOX11* plants, whereas the ROS burst was strongly delayed in *oscox11*/*OsCOX11* cells after chitin treatment (Fig. 5a). Staining with 3, 3′-diaminobenzidine (DAB) also showed that pathogen-triggered ROS accumulation was markedly reduced in *P*_*Ubi*_*::Avr-Pita* and *P*_*ubi*_*::OsCOX11* *vs.* WT plants (Fig. 5b). These results indicate that Avr-Pita and OsCOX11 function in pathogen infection by suppressing ROS accumulation.

To investigate whether Avr-Pita manipulates OsCOX11-mediated ROS metabolism at the transcriptional level, we measured the expression of *OsCOX11* in plants after chitin treatment and fungal infection. The expression of *OsCOX11* did not significantly change after chitin treatment in *P*_*Ubi*_*::Avr-Pita* or WT plants (Fig. S5a). And the expression of *OsCOX11* was induced slightly only at 6 h post *M. oryzae* infection (Fig. S5b). These results suggest that Avr-Pita does not significantly promote *OsCOX11* at the transcriptional level.

**Fig. 6:**
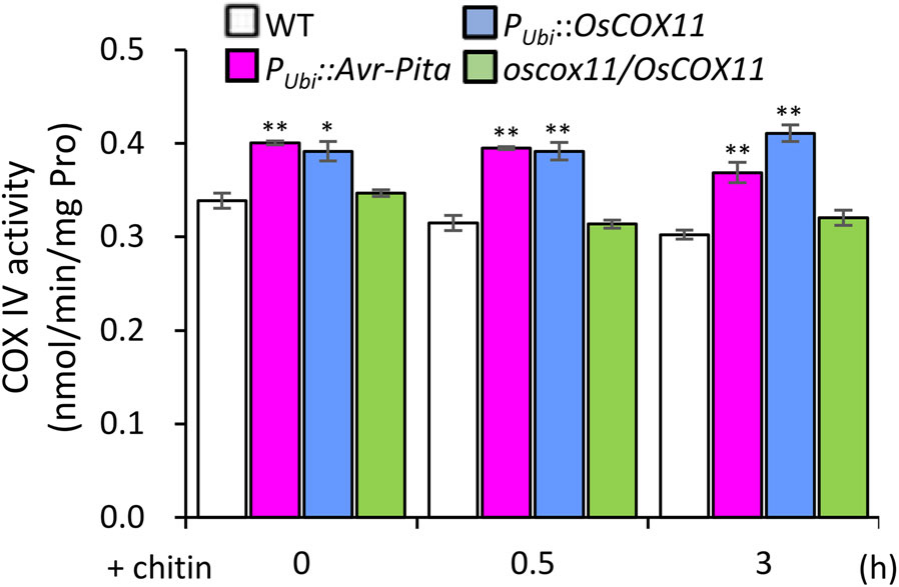
Avr-Pita promotes cytochrome *c* oxidase activity. COX activity was measured in WT, *P*_*Ubi*_*::Avr-Pita*, *P*_*Ubi*_*::OsCOX11*, and *oscox11/OsCOX11* plants following chitin treatment. Data are shown as mean ± SD (**P < 0.01, *P < 0.05, *n* = 3)

Given that COX assembly proteins are present in METC Complex IV, we examined whether the interaction of Avr-Pita with COX11 affects COX activity. COX activity was much higher in *P*_*Ubi*_*::Avr-Pita* and *P*_*Ubi*_*::OsCOX11* plants than in the WT both before and after chitin treatment (Fig. 6). These results suggest that the effect of *Avr-Pita* overexpression is equivalent to that of *OsCOX11*, as overexpressing either gene promoted COX activity to reduce ROS accumulation.

**Fig. 7:**
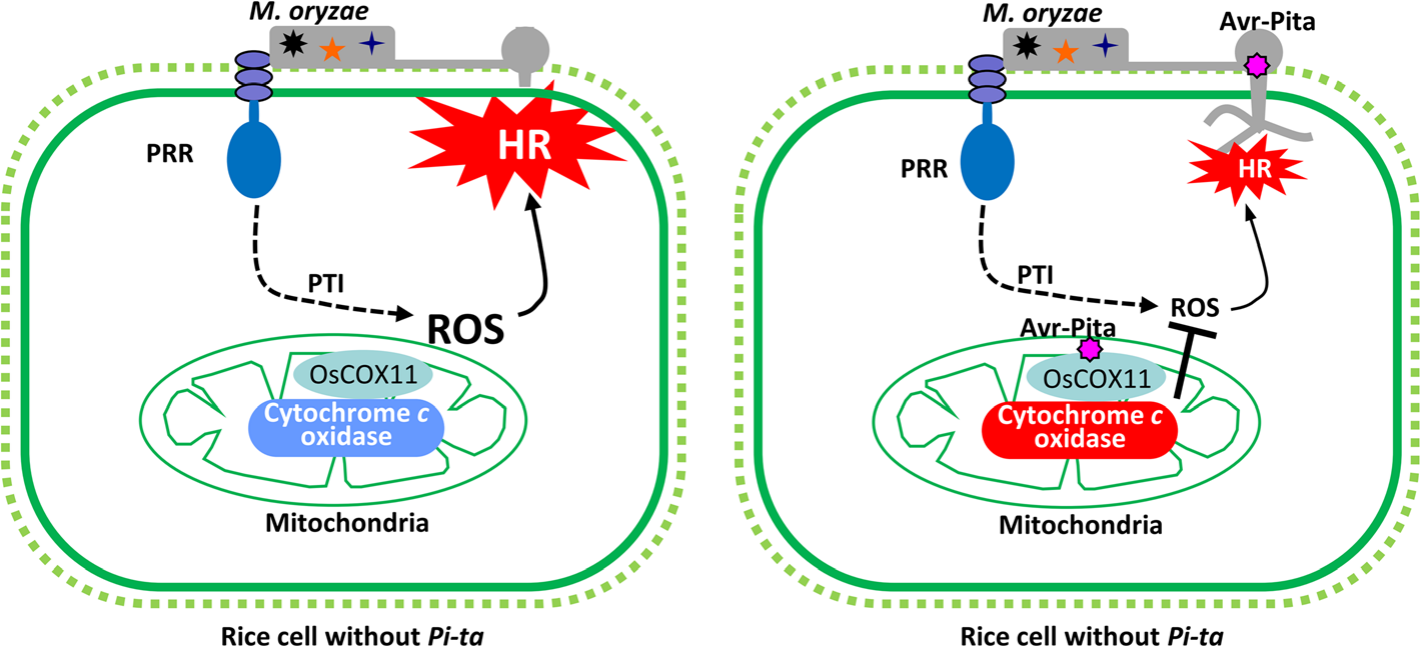
A working model for the role of Avr-Pita in regulating defense responses in rice. The PRR on the rice plasma membrane perceives PAMP molecules from the fungal pathogen (*M. oryzae*) and activates PTI resulting in a ROS burst and HR to stop microbial invasion (left). In *M. oryzae* isolates carrying the effector Avr-Pita (right), this effector is secreted from *M. oryzae* and delivered into rice cells. There, Avr-Pita targets the host mitochondria and binds to OsCOX11 to promote COX activity, thereby decreasing ROS accumulation, resulting in reduced resistance to *M. oryzae*

Based on these results, we propose a working model for the role of the effector Avr-Pita in disturbing host innate immunity. According to our model, *M. oryzae* secretes Avr-Pita into rice cells through the BIC structure of invasive hyphae during rice cell invasion. Avr-Pita targets the host mitochondria and interacts with the COX assembly protein OsCOX11 to promote COX activity, leading to reduced ROS accumulation and increased susceptibility to the pathogen (Fig. 7).

## Discussion

During plant–pathogen interactions, the pathogens deliver a number of effectors into the host cells to disturb host innate immunity and facilitate their own propagation (Zhang & Xu, 2014). In-depth studies of bacteria–plant interactions have revealed that bacterial pathogens manipulate host immunity *via* their effectors (Deslandes & Rivas, 2012). By contrast, little is known about the roles of fungal effectors in this process. For example, the localization and intrinsic function of Avr-Pita in host cells have remained long-standing questions following the discovery of the Avr-Pita/Pi-ta fungal pathosystem in rice (Orbach et al., 2000). In the current study, we found that Avr-Pita suppresses chitin-triggered ROS accumulation and *PR* gene expression (Figs. 1d–e, 5a and S1f) and enhances susceptibility to *M. oryzae* in rice (Fig. 1a–c).

Pathogen effectors can be divided into apoplastic and intracellular effectors based on their localization. Most bacterial effectors and some fungal effectors are dispersed in the apoplastic space, such as the *M. oryzae* effector Slp1 (Mentlak et al., 2012), the *Ustilago maydis* effectors Pep1 (Doehlemann et al., 2009) and Pit2 (Mueller et al., 2013), and the *Cladosporium fulvum* effectors Avr2 (Rooney et al., 2005) and Avr4 (van den Burg et al., 2006). Intracellular effectors are secreted by invasive hyphae and delivered into host cells. The fungal effectors PWL1 and PWL2 (Khang et al., 2010), AvrPiz-t (Park et al., 2012), and Avr-Pii (Singh et al., 2016) initially accumulate in the BIC structure. After they enter the host cells, PWL2 (Khang et al., 2010), AvrPiz-t (Park et al., 2012), and Avr1-CO39 (Ribot et al., 2013) localize to the cytoplasm, and Avr-Pib partially targets the host nucleus (Zhang et al., 2018). Avr-Pita also accumulates in the BIC structure of *M. oryzae* during rice cell invasion (Khang et al., 2010). In the current study, we demonstrated that Avr-Pita targets the rice mitochondria (Fig. 3a and b, S3a and S3b) and interacts with OsCOX11 to regulate mitochondrial COX activity (Fig. 2a and b). Avr-Pita specifically binds to the region of 2th β sheet to 5th β sheet of the OsCOX11. Especially, the 5th β sheet is critical for the interaction, indicating that Avr-Pita may modify the specific residues in 5th β sheet of OsCOX11 as a metalloprotease to promotes the COX activity. The heterozygous *oscox11*/*OsCOX11* knockout mutants, in which the *OsCOX11* expression reduced ~ 50%, become resistant to the fungal pathogen (Fig. 4d and e). However, the sensitivity of current method for COX activity measurement may be too low to detect the expected COX activity reduction in the *oscox11*/*OsCOX11* plants (Fig. S4c).

ROS such as H_2_O_2_, O_2_^·−^, and ^1^O_2_ play critical roles in plant immunity. In response to the perception of pathogen invasion, plants activate a rapid ROS burst and induce the HR *via* an immune signaling cascade (Kou et al., 2019). Apoplastic ROS production mediated by plasma membrane NADPH oxidases or cell wall peroxidases provides a major source of ROS for plant defense responses (Kadota et al., 2015). During plant–pathogen interactions, pathogens secrete a number of effectors into the host apoplast and cytoplasm. These apoplastic effectors interfere with PAMP perception mediated by plant membrane-bound pattern recognition receptor (PPR), leading to the inactivation of NADPH oxidases. By contrast, cytoplasmic effectors target the MAPK signaling pathway, vesicle trafficking, and metabolic priming, which are essential for apoplastic ROS production (Jwa & Hwang, 2017). Chloroplast-generated ROS also contribute to plant defense responses. The photosynthetic electron transport chains of photosystem I (PSI) and photosystem II (PSII) are two main sources of ROS production in chloroplasts (Lu & Yao, 2018). Several bacterial effectors such as HopNI and HopK1 target the chloroplast and manipulate chloroplast function associated with ROS production (Kretschmer et al., 2019). The fungal effector ToxA induces plant ROS accumulation in chloroplasts, which leads to cell death (Manning et al., 2009). The accumulation of ROS is light dependent and is associated with the dysfunction of the PSI and PSII complexes (Manning et al., 2009).

Mitochondria are important organelles for energy generation and ROS production (Jacoby et al., 2012; Mackenzie & McIntosh, 1999). Mitochondrial ROS, a major source of ROS in plant cells, is produced by Complexes I, II, and III of the METC, while the terminal complex (Complex IV) functions in ROS consumption *via* the COX-mediated transfer of electrons to molecular oxygen, eventually forming H_2_O (Timon-Gomez et al., 2018). Mitochondrial oxidase of the respiratory chain is a determinant of ROS generation involved in pathogen defense responses (Cvetkovska & Vanlerberghe, 2013; Vidal et al., 2007). The cytoplasmic male sterility protein WA352 interacts with OsCOX11 and inhibits its function in ROS scavenging, thereby inducing tapetal PCD in rice (Luo et al., 2013). Decreasing *AtCOX11* expression suppresses COX activity in *Arabidopsis*, resulting in abnormal plant growth and pollen germination (Radin et al., 2015). However, no pathogen effectors have been shown to target mitochondria and interfere with mitochondrial ROS metabolism. In the current study, we demonstrated that silencing *OsCOX11* limits pathogen propagation (Fig. 4d–f and S4e–S4g), whereas overexpressing this gene inhibits ROS accumulation and enhances susceptibility to *M. oryzae* (Figs. 4a–c and 5a–c), indicating that COX-mediated ROS metabolism is involved in plant resistance to this fungus. Furthermore, overexpressing *Avr-Pita* increased COX activity (Fig. 6) and suppressed ROS accumulation (Fig. 5) in rice, suggesting that the pathogen delivers the effector Avr-Pita to suppress host immune responses by enhancing OsCOX11-dependent ROS metabolism (Fig. 7).

## Conclusions

Pathogens produce effectors to hinder host defenses, such as defense gene expression, or the production of ROS, which act as signaling molecules and (at higher concentrations) can directly kill pathogens. Avr-Pita was the first fungal effector protein identified from *M. oryzae*, the devastating fungal pathogen that causes rice blast disease, but its role in this plant–pathogen interaction is poorly understood. In current study, we provide evidence that Avr-Pita targets rice mitochondria and interacts with the COX assembly protein OsCOX11 of the METC. This interaction promotes the activity of COX to decrease ROS accumulation, thereby suppressing innate immunity in rice. This novel finding increases our understanding of how a pathogen effector dampens host innate immunity by hijacking a plant protein and manipulating mitochondrial activity in the *M. oryzae*–rice interaction.

## Material and Methods

### Generation and Characterization of Transgenic Plants

To generate transgenic rice lines with estradiol-inducible expression of *Avr-Pita*, a truncated fragment* Avr-Pita*^*145–672*^ encoding a mature protease was isolated and subcloned into the estradiol-inducible binary expression vector pXVE. The resulting construct (*P*_*XVE*_*::Avr-Pita*) was transformed into *rice *variety Kinmaze (without* Pi-ta*). To generate transgenic rice plants with stable overexpression of* Avr-Pita *or* OsCOX11*, the* Avr-Pita*^*145–672*^ fragment and full-length coding sequence of *OsCOX11 *were individually inserted into the binary vector pOX (containing the maize *Ubiquitin* promoter) to generate *the P*_*Ubi*_*::Avr-Pita* and *P*_*Ubi*_*::OsCOX11* constructs, which were introduced into the rice cultivar Nipponbare (without* Pi-ta*).

Knockout of *OsCOX11* in rice cultivar Nipponbare was performed by CRISPR/Cas9 gene editing as described previously (Ma et al., 2015). In brief, the 20-bp target site of *OsCOX11* (5′-TTTAATGCTGACGTTGCTGA-3′) driven by the *U6b* promoter was cloned into the binary vector pYLCRISPR-Cas9Pubi-H. To silence the expression of *OsCOX11* in the rice cultivar Tohoku IL6 background (carrying* Pi-ta*), ~ 0.35-kb sense and antisense fragments of the *OsCOX11* coding sequence were cloned into the binary vector *P*_*OsCERK1*_-RNAi (driven by the *OsCERK1* promoter) to produce the *P*_*OsCERK1*_*::OsCOX11-RNAi* construct.

The genotypes of *OsCOX11* knockout plants were investigated by PCR using primer pair OsCOX11-In2Ex4-F/OsCOX11-In2Ex4-R, followed by sequencing with the OsCOX11-seq-F primer (Table S2). To measure *Avr-Pita* and *OsCOX11* transcript levels in the transgenic plants, leaf blades were sampled from T_0_ or T_1_ plants and immediately frozen in liquid nitrogen for RNA extraction. Gene expression was then measured as described below.

### Pathogen Inoculation and Evaluation of Disease Resistance

*M. oryzae* isolates 10–441 and 13–219, which are virulent on rice varieties Nipponbare and Tohoku IL6, were used to assess the resistance of rice to blast disease. The isolates were grown on Potato Dextrose Agar medium, and conidial production was induced on Oatmeal Agar medium under 12-h dark/12-h blue-light irradiation.

For leaf spray inoculation, three- to four-week-old seedlings were spray-inoculated with spore suspension (0.5 × 10^5^ spores/mL, containing 0.01% Tween 20). The inoculated plants were maintained in a growth chamber at 26 °C with 90% humidity in the dark for 24 h, followed by incubation under a 12 h light/12 h dark cycle. The inoculated leaves were sampled at 0, 6, 12, 24, and 48 h for gene expression analysis.

For leaf punch inoculation, the young leaves of six- to eight-week-old plants were wounded with a mouse ear puncher. A piece of a *M. oryzae* spore colony was affixed to the wounding site and sealed with transparent tape. The inoculated leaves were collected and photographed at 12 dpi. The size of the lesion area was quantified using Image J software. Relative fungal biomass in infected leaves was measured by DNA-based quantitative PCR using *MoPot2-* and *OsUbq-*specific primers (Table S2) and determined using the eq. 2^[CT(*OsUbq*)-CT(*MoPot2*)]^ (Berruyer et al., 2006).

To inoculate rice leaf sheaths, ~ 5-cm leaf sheath samples were collected from four- to six-week-old plants and injected with fungal spores (0.5 × 10^5^ spores/mL, in 0.2% gelatin) in their hollow interiors. The infected samples were incubated in a 90% humidity chamber at 26 °C in the dark, and ROS detection was conducted at 0, 24, 48, and 96 h.

### Gene Expression Analysis

To investigate the expression levels of defense response genes in different transgenic lines, leaf discs or suspension cells were treated with 200 μg/mL chitin and sampled at 0, 3, and 6 h. Suspensions cells derived from *P*_*XVE*_*::Avr-Pita* transgenic lines were treated with estradiol and sampled at 0, 3, and 6 h for expression analysis.

Total RNA was extracted from various rice tissues using TRIzol reagent (Thermo Fisher Scientific), and gDNA was removed using RNase-free RQ1 DNaseI (Promega). First-strand cDNA was synthesized with oligonucleotide dT primers and M-MLV reverse transcriptase (Promega). Transcript levels were quantified by qRT-PCR with gene-specific primers (Table S2) using SYBR Green master mix (Bio-Rad) on a CFX96 Real-Time System (Bio-Rad). The relative expression level of each gene was normalized to that of *OsACTIN* using the 2^−ΔCt^ method.

### Yeast Two-Hybrid Assays

The interactions between Avr-Pita and various COX11 truncated proteins were analyzed using the GAL4 Y2H system (Clontech). The *Avr-Pita*^*145–672*^ fragment was cloned into bait vector pGBKT7 (BD). Full-length fragments of the *OsCOX11*, *AtCOX11*, *ScCOX11*, and *MoCOX11* coding sequences were amplified from the corresponding organisms and subcloned separately into the prey vector pGADT7 (AD). A series of truncated *OsCOX11* prey vectors including *OsCOX11*^*1–198*^, *OsCOX11*^*80–220*^, *OsCOX11*^*110–220*^, and *OsCOX11*^*140–244*^ were generated based on the *OsCOX11* prey vector using the Ω-PCR method (Chen et al., 2013).

Various combinations of the bait and prey constructs were co-transformed into yeast Y2H Gold cells using the Matchmaker Gold Yeast Two-Hybrid System (Clontech). The transformants were grown on synthetic dropout medium without Leucine and Tryptophan (SD-LW) and synthetic dropout medium lacking Adenine, Histidine, Leucine, and Tryptophan (SD-AHLW) at 30 °C for 3 days.

### Pull-Down Assays

The *GST:Avr-Pita* and *His:OsCOX11* plasmids used for the pull-down assay were generated using the Ω-PCR method. Briefly, the *Avr-Pita*^*145–672*^ fragment and full-length* OsCOX11 *were individually subcloned into pGEX-4 T-2 (containing GST tag) (Sigma) and the prokaryotic expression vector pET32a (containing N-Trx, N-6×His, N-thrombin, N-S, N-enterokinase, C-6×His tag) (Novagen) by Ω-PCR (Chen et al., 2013).

The fusion proteins were expressed in *E. coli* BL21 cells, purified using GST or HIS binding resin, and subjected to pull-down assays. The pulled-down proteins were subjected to SDS-PAGE and transferred to nitrocellulose membranes. Specific anti-GST and anti-His antibodies and HRP-conjugated antibody were used for immunoblot analysis. The signals were visualized with Immobilon Western HRP substrate and detected by automatic chemiluminescence image analysis (Tanon 5200 Multi).

### Subcellular Localization of Fusion Proteins

Subcellular localization analysis of various fusion proteins was performed using transient expression vectors *Avr-Pita:YFP* and *OsCOX11:mCherry* carrying the *Avr-Pita*^*145–672*^ fragment and full-length cDNA of *OsCOX11*, respectively, driven by the 35S promoter.

The subcellular localization of Avr-Pita:YFP and OsCOX11:mCherry was investigated in rice protoplasts or onion epidermal cells. For transient expression in rice protoplasts, the indicated plasmids were co-transfected into rice protoplasts using PEG/Ca-mediated transfection and incubated for 12–18 h at 28 °C in the dark. The plasmids were also introduced into onion epidermal cells using a DNA particle delivery system (Bio-Rad, Helios Gene Gun). Following bombardment, the onion epidermal cells were incubated on Murashige and Skoog medium for 16–20 h at 28 °C in the dark. The transfected rice protoplasts or onion epidermal cells were observed under a laser-scanning confocal microscope (Carl Zeiss, LSCM 7DUO) with excitation laser wavelengths of 514 nm (YFP) and 543 nm (mCherry).

### Detection of Reactive Oxygen Species

To measure ROS accumulation using the luminol-chemiluminescence method, rice suspension cell cultures containing 50 μM L-012, 2 mg/mL horseradish peroxidase, and 20 mg/mL chitin ((GlcNAc)_8_) (distilled water was used as a control) were introduced into 96-well plates. Luminescence was immediately measured at 30-s intervals for 120 min using a Multi-Mode Microplate Reader (BioTek, SynergyMx). Four biological replications were performed for each sample.

To detect the ROS using DAB staining, fungal-infected rice leaf sheaths were submerged in 1 mg/mL DAB solution (pH 3.8), and chlorophyll was completely removed from the samples with 95% boiling ethanol. The DAB-stained ROS appeared reddish-brown. The stained leaf sheaths were photographed under an optical microscope (Carl Zeiss, Axio Observer Z1).

### Measuring Cytochrome *c* Oxidase Activity

Mitochondrial protein was extracted from rice tissues for COX activity measurements using a Mitochondrial Complexes IV Assay Kit (Suzhou Keming biotechnology) according to the manufacturer’s protocol. Briefly, chitin-treated (200 μg/mL) rice tissues were ground in liquid nitrogen and homogenized in ice-cold Extraction Buffer containing protease inhibitor. The supernatants were harvested by centrifugation, and COX activity in each sample was measured with a Multi-Mode Microplate Reader (BioTek, SynergyMx).

## Supplementary Information

**Additional file 1: **Figure S1 Ectopic expression of ***Avr-Pita*** in rice.** (a)** Diagram of the *P*_*XVE*_*::Avr-Pita* and *P*_*Ubi*_*::Avr-Pita* constructs. In these constructs, a truncated *Avr-Pita*^*145–672*^ fragment encoding mature protease is driven by the estradiol-inducible promoter *XVE* or the maize *Ubiquitin* (*Ubi*) promoter. SP: signal peptide; Pro: predicted prosequence. ***(b)*** The growth and developmental morphology of *P*_*Ubi*_*::Avr-Pita* plants were not obviously affected in these lines. Scale bars: 10 cm. **(c)** Transcript levels of *Avr-Pita* was measured by RT-PCR in *P*_*XVE*_::*Avr-Pita *transgenic suspension cell lines after estradiol treatment*.* **(d)** Transcript levels of *Avr-Pita* was measured by RT-PCR in *P*_*Ubi*_::*Avr-Pita* transgenic lines. **(e)** The expression of* Avr-Pita *in *P*_*XVE*_::*Avr-Pita *transgenic suspension cell lines after estradiol treatment using qRT-PCR*.* ***(f)*** The expression of defense-response gene* OsPAL1 *in *P*_*XVE*_::*Avr-Pita *suspension cell lines after estradiol treatment using qRT-PCR. ***(g)*** Overexpression of *Avr-Pita* in *P*_*Ubi*_::*Avr-Pita* transgenic lines using qRT-PCR. *OsACTIN* served as a control to normalize the expression levels of target genes. Data are shown as mean ± SD (**P < 0.01, *n* = 3). **Figure S2** Avr-Pita specifically binds to the conserved domains of OsCOX11. **(a)** Amino acid sequence alignment of COX11 orthologs OsCOX11 (*O. sativa*, XP_006650503.1), AtCOX11 (*A. thaliana*, AAG00893), ScCOX11 (*S. cerevisiae*, NP_015193), and MoCOX11 (*M. oryzae*, XP_003717808). Six β sheets (indicated by a single underline and numbered 1–6) and Cu-binding core region CFCF (indicated by four triangles ▲) are present in the conserved region of COX11. Residues 140–220 of OsCOX11, which are responsible for the interaction with Avr-Pita, are double underlined. The critical region OsCOX11^199–220^ is labelled with boxes. **(b)** Avr-Pita specifically interacts with rice OsCOX11 in a Y2H assay. Yeast cells were cultured on selective medium SD-LW or SD-AHLW; cell growth on SD-AHLW indicates a positive interaction. **Figure S3** Avr-Pita and OsCOX11 co-localize to the mitochondria in onion epidermal cells. **(a)** *Avr-Pita:YFP* was introduced into onion epidermal cells by particle bombardment and stained with the mitochondrial dye MitoTracker. **(b)** *Avr-Pita:YFP* and *OsCOX11:mCherry* were transiently introduced into onion epidermal cells by particle bombardment. Scale bar = 50 μm. **Figure S4** Characterization of *OsCOX11* transgenic plants and pathogen resistance of *OsCOX11*-RNAi plants. **(a)** Expression levels of *OsCOX11* in *P*_*Ubi*_::*OsCOX11* lines; *OsACTIN* served as an internal control. Data are shown as mean ± SD (**P < 0.01, *n* = 3). **(b)** Genotypes of heterozygous *oscox11/OsCOX11* lines carrying a “T” or “C” base insertion. **(c)** Expression levels of *OsCOX11* in *oscox11/OsCOX11* plants. *OsACTIN* served as an internal control. Data are shown as mean ± SD (**P < 0.01, *n* = 3). **(d)** Expression levels of *OsCOX11* in *OsCOX11*-RNAi plants. *OsACTIN* served as an internal control. Data are shown as mean ± SD (**P < 0.01, *n* = 3). **(e)** Disease symptoms of *OsCOX11*-RNAi transgenic plants at 12 dpi inoculated with *M. oryzae* isolate 13–219. **(f)** Lesion area in *OsCOX11*-RNAi transgenic plants at 12 dpi inoculated with *M. oryzae* isolate 13–219. Data are shown as mean ± SD (**P < 0.01, *n* > 12). **(g)** Relative fungal biomass on inoculated leaves at 12 dpi, as determined by qPCR. Data are shown as mean ± SD (**P < 0.01, *n* = 3). **Figure S5** *OsCOX11* expression in response to chitin and *M. oryzae* treatment. **(a)** The expression levels of *OsCOX11* in *P*_*Ubi*_::*Avr-Pita* and WT plants after chitin treatment. **(b)** The expression levels of *OsCOX11* in rice seedlings in response to *M. oryzae* compatible strain 08-T13 inoculation at the indicated time points. *OsACTIN* served as an internal control. Data are shown as mean ± SD (**P < 0.01, *n* = 3). **Table S1 CC**andidates of Avr-Pita interacting protein screened by Y2H. **Table S2** Primers used in this study.

## Data Availability

All relevant data are provided within the article and its supplementary information files.

## Abbreviations

Avr: Avirulence
BIC: Biotrophic interfacial complex
COX: Cytochrome *c* oxidase
ETI: Effector-triggered immunity
HR: Hypersensitive response
MAPK: Mitogen activated protein kinase
METC: Mitochondrial electron transport chain
NBS-LRR: Nucleotide binding site-leucine rich repeat domain
PAMP: Pathogen-associated molecular pattern
PCD: Programmed cell death
PR: Pathogenesis-related
PTI: PAMP-triggered immunity
PSI: Photosystem I
PSII: Photosystem II
ROS: Reactive oxygen species
