# Supplementary material for: The Fungal Effector Avr-Pita Suppresses Innate Immunity by Increasing COX Activity in Rice Mitochondria
Source: Rice (N Y). 2021 Jan 14;14:12. doi: 10.1186/s12284-021-00453-4 (PMC7809080; doi:10.1186/s12284-021-00453-4)
Supplement: Supplementary file 1 — Additional file 1: Figure S1 Ectopic expression of Avr-Pita in rice. (a) Diagram of the PXVE::Avr-Pita and PUbi::Avr-Pita constructs. In these constructs, a truncated Avr-Pita145–672 fragment encoding mature protease is driven by the estradiol-inducible promoter XVE or the maize Ubiquitin (Ubi) promoter. SP: signal peptide; Pro: predicted prosequence. (b) The growth and developmental morphology of PUbi::Avr-Pita plants were not obviously affected in these lines. Scale bars: 10 cm. (c) Transcript levels of Avr-Pita was measured by RT-PCR in PXVE::Avr-Pita transgenic suspension cell lines after estradiol treatment. (d) Transcript levels of Avr-Pita was measured by RT-PCR in PUbi::Avr-Pita transgenic lines. (e) The expression of Avr-Pita in PXVE::Avr-Pita transgenic suspension cell lines after estradiol treatment using qRT-PCR. (f) The expression of defense-response gene OsPAL1 in PXVE::Avr-Pita suspension cell lines after estradiol treatment using qRT-PCR. (g) Overexpression of Avr-Pita in PUbi::Avr-Pita transgenic lines using qRT-PCR. OsACTIN served as a control to normalize the expression levels of target genes. Data are shown as mean ± SD (**P < 0.01, n = 3). Figure S2 Avr-Pita specifically binds to the conserved domains of OsCOX11. (a) Amino acid sequence alignment of COX11 orthologs OsCOX11 (O. sativa, XP_006650503.1), AtCOX11 (A. thaliana, AAG00893), ScCOX11 (S. cerevisiae, NP_015193), and MoCOX11 (M. oryzae, XP_003717808). Six β sheets (indicated by a single underline and numbered 1–6) and Cu-binding core region CFCF (indicated by four triangles ▲) are present in the conserved region of COX11. Residues 140–220 of OsCOX11, which are responsible for the interaction with Avr-Pita, are double underlined. The critical region OsCOX11199–220 is labelled with boxes. (b) Avr-Pita specifically interacts with rice OsCOX11 in a Y2H assay. Yeast cells were cultured on selective medium SD-LW or SD-AHLW; cell growth on SD-AHLW indicates a positive interaction. Figure S3 Avr-P [file 12284_2021_453_MOESM1_ESM.pdf]

Supplementary Information

The Fungal Effector Avr-Pita Suppresses Innate Immunity by Increasing COX Activity in Rice Mitochondria

Jingluan Han[1, 2, 3 #], Xiaoyu Wang[1, 3 #], Fengpin Wang[1, 3 #], Zhe Zhao[1, 2, 3], Gousi Li[1, 3], Xiaoyuan Zhu[4], Jing Su[4 *], and Letian Chen[1, 2, 3 *]

Supplementary Figures

Fig. S1 Ectopic expression of *Avr-Pita* in rice.

Fig. S2 Avr-Pita specifically binds to the conserved domains of OsCOX11.

Fig. S3 Avr-Pita and OsCOX11 co-localize to the mitochondria in onion epidermal cells.

Fig. S4 Characterization of *OsCOX11* transgenic plants and pathogen resistance of *OsCOX11*-RNAi plants.

Fig. S5 *OsCOX11* expression in response to chitin and *M. oryzae* treatment.

Supplementary Tables

Table. S1 Candidates of Avr-Pita interacting protein screened by Y2H.

Table. S2 Primers used in this study.

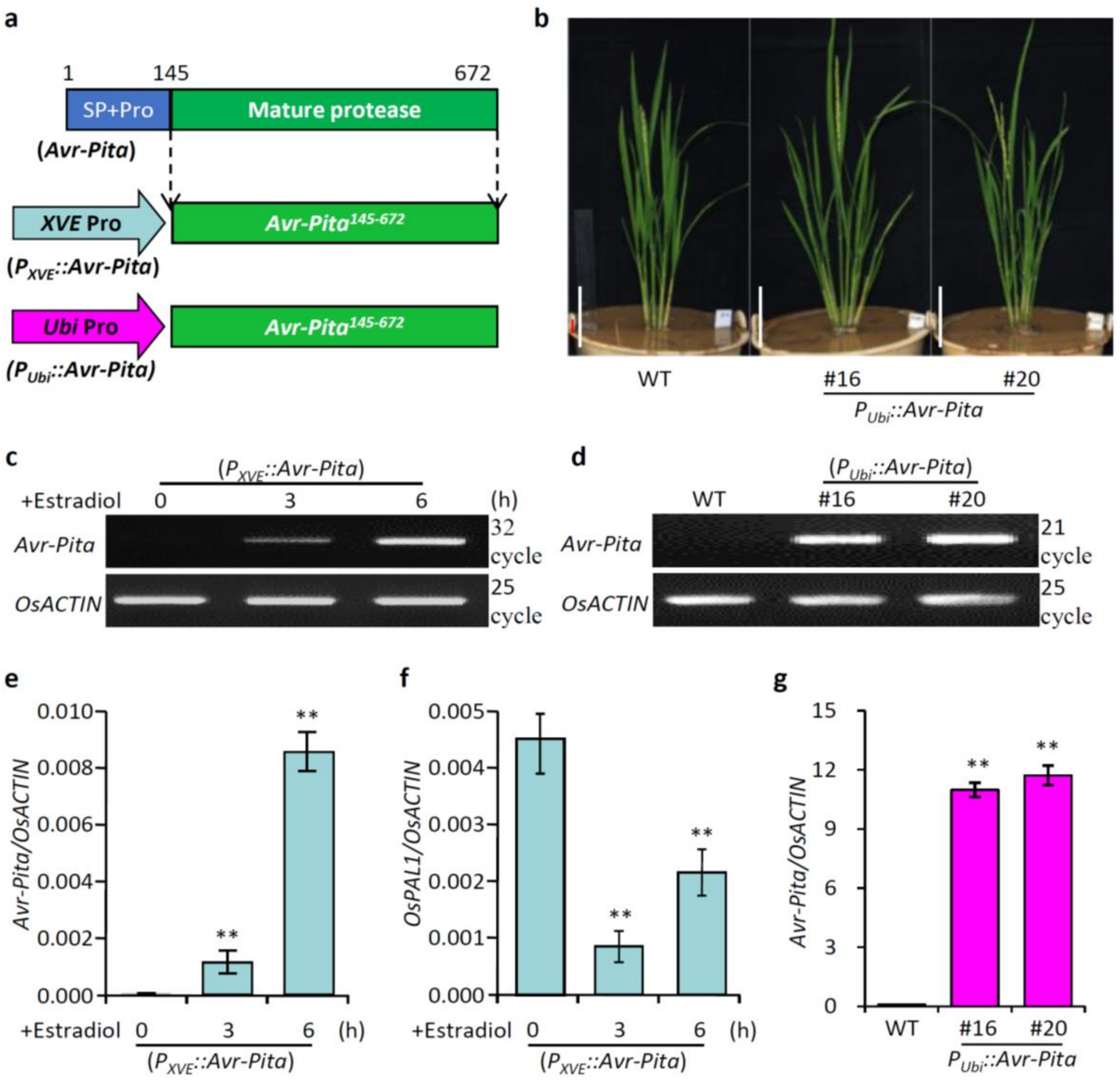

Fig. S1 Ectopic expression of *Avr-Pita* in rice.

(a) Diagram of the P_{XVE}::Avr-Pita and P_{Ubi}::Avr-Pita constructs. In these constructs, a truncated *Avr-Pita*$^{145–672}$ fragment encoding mature protease is driven by the estradiol-inducible promoter *XVE* or the maize *Ubiquitin* (*Ubi*) promoter. SP: signal peptide; Pro: predicted prosequence. **(b)** The growth and developmental morphology of P_{ubi}*::Avr-Pita* plants were not obviously affected in these lines. Scale bars: 10 cm. **(c)** Transcript levels of *Avr-Pita* was measured by RT-PCR in P_{XVE}*::Avr-Pita* transgenic suspension cell lines after estradiol treatment. **(d)** Transcript levels of *Avr-Pita* was measured by RT-PCR in P_{ubi}*::Avr-Pita* transgenic lines. **(e)** The expression of *Avr-Pita* in P_{XVE}*::Avr-Pita* transgenic suspension cell lines after estradiol treatment using qRT-PCR. **(f)** The expression of defense-response gene *OsPAL1* in P_{XVE}*::Avr-Pita* suspension cell lines after estradiol treatment using qRT-PCR. **(g)** Overexpression of *Avr-Pita* in P_{ubi}*::Avr-Pita* transgenic lines using qRT-PCR. *OsACTIN* served as a control to normalize the expression levels of target genes. Data are shown as mean ± SD (**$P < 0.01$, $n = 3$).

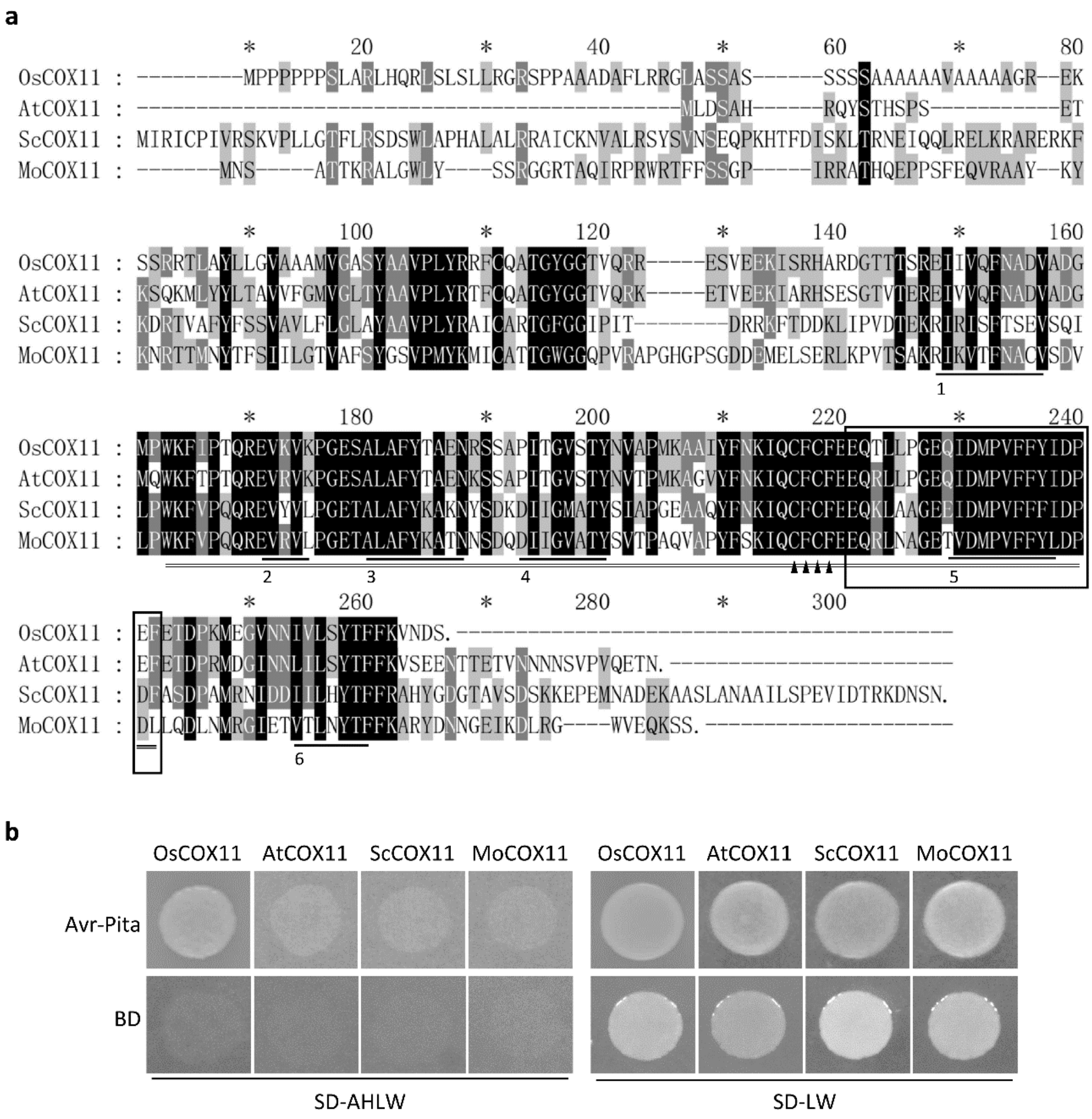

Fig. S2 Avr-Pita specifically binds to the conserved domains of OsCOX11.

(a) Amino acid sequence alignment of COX11 orthologs OsCOX11 (*O. sativa*, XP_006650503.1), AtCOX11 (*A. thaliana*, AAG00893), ScCOX11 (*S. cerevisiae*, NP_015193), and MoCOX11 (*M. oryzae*, XP_003717808). Six β sheets (indicated by a single underline and numbered 1–6) and Cu-binding core region CFCF (indicated by four triangles ▲) are present in the conserved region of COX11. Residues 140–220 of OsCOX11, which are responsible for the interaction with Avr-Pita, are double underlined. The critical region OsCOX11$^{199–220}$ is labelled with boxes. **(b)** Avr-Pita specifically interacts with rice OsCOX11 in a Y2H assay. Yeast cells were cultured on selective medium SD-LW or SD-AHLW; cell growth on SD-AHLW indicates a positive interaction.

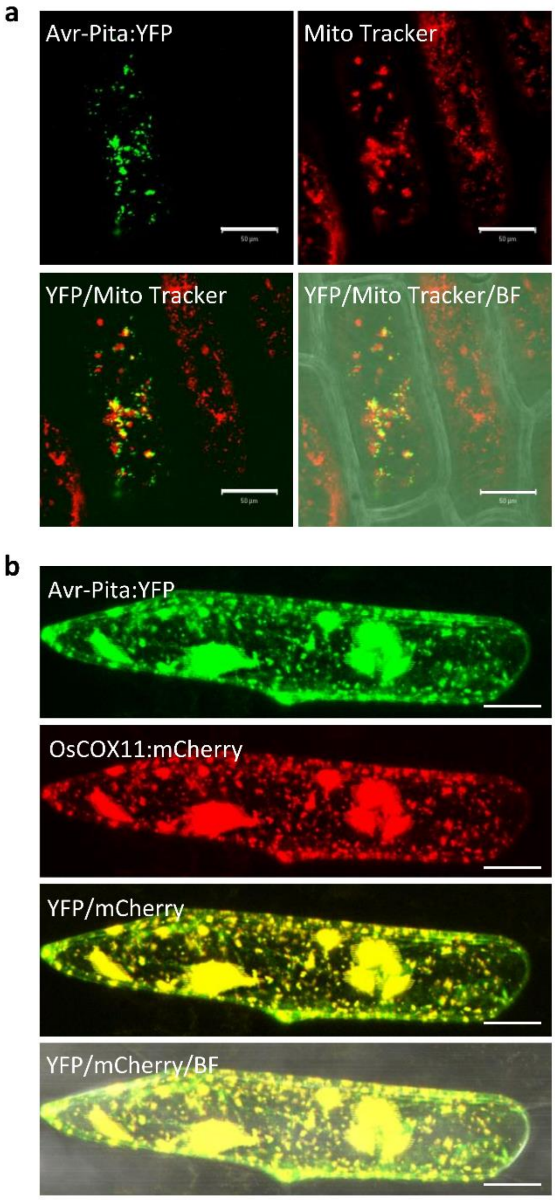

Fig. S3 Avr-Pita and OsCOX11 co-localize to the mitochondria in onion epidermal cells.

(a) *Avr-Pita:YFP* was introduced into onion epidermal cells by particle bombardment and stained with the mitochondrial dye MitoTracker. **(b)** *Avr-Pita:YFP* and *OsCOX11:mCherry* were transiently introduced into onion epidermal cells by particle bombardment. Scale bar = 50 μm.

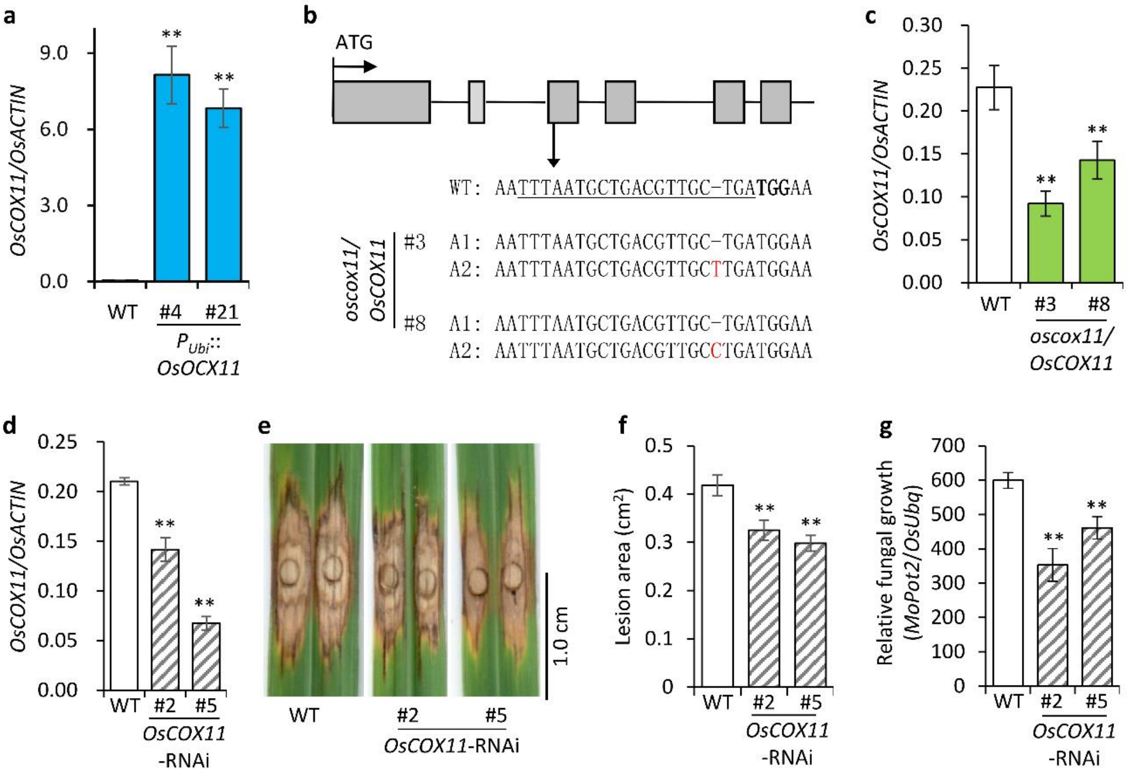

Fig. S4 Characterization of *OsCOX11* transgenic plants and pathogen resistance of *OsCOX11*-RNAi plants.

(a) Expression levels of *OsCOX11* in P_{Ubi}::*OsCOX11* lines; *OsACTIN* served as an internal control. Data are shown as mean ± SD (**P < 0.01, n = 3). **(b)** Genotypes of heterozygous *oscox11*/*OsCOX11* lines carrying a "T" or "C" base insertion. **(c)** Expression levels of *OsCOX11* in *oscox11*/*OsCOX11* plants. *OsACTIN* served as an internal control. Data are shown as mean ± SD (**P < 0.01, n = 3). **(d)** Expression levels of *OsCOX11* in *OsCOX11*-RNAi plants. *OsACTIN* served as an internal control. Data are shown as mean ± SD (**P < 0.01, n = 3). **(e)** Disease symptoms of *OsCOX11*-RNAi transgenic plants at 12 dpi inoculated with *M. oryzae* isolate 13-219. **(f)** Lesion area in *OsCOX11*-RNAi transgenic plants at 12 dpi inoculated with *M. oryzae* isolate 13-219. Data are shown as mean ± SD (**P < 0.01, n >12). **(g)** Relative fungal biomass on inoculated leaves at 12 dpi, as determined by qPCR. Data are shown as mean ± SD (**P < 0.01, n = 3).

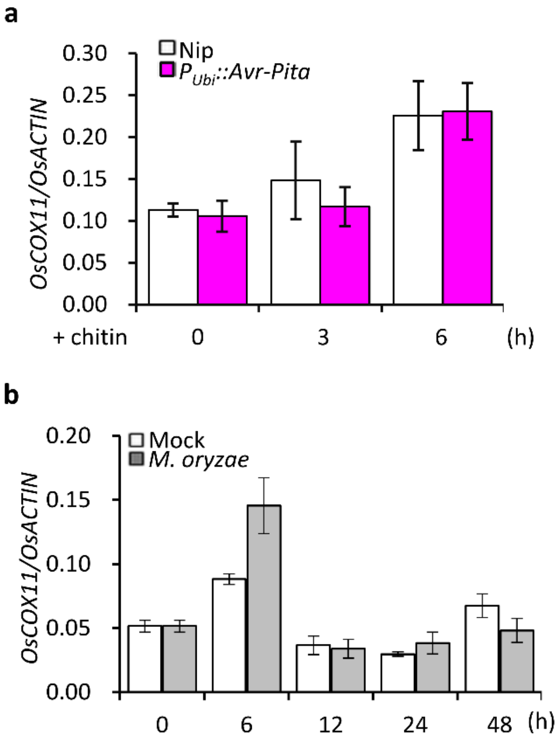

Fig. S5 *OsCOX11* expression in response to chitin and *M. oryzae* treatment.

(a) The expression levels of *OsCOX11* in P_{Ubi}*::Avr-Pita* and WT plants after chitin treatment. **(b)** The expression levels of *OsCOX11* in rice seedlings in response to *M. oryzae* compatible strain 08-T13 inoculation at the indicated time points. *OsACTIN* served as an internal control. Data are shown as mean ± SD (**$P < 0.01$, $n = 3$).

Table. S1 Candidates of Avr-Pita interacting protein screened by Y2H.

Gene ID	Predicted Function
Os01g0127500	Dihydroflavonol-4-reductase
Os01g0531500	Dienelactone hydrolase family protein
Os03g0718600	Cytochrome c oxidase assembly protein COX11
Os08g0532900	Emp24/gp25L/p24 family protein
Os06g0149900	Cysteine synthase
Os11g0171300	Fructose-bisphospate aldolase isozyme

Table. S2 Primers used in this study.

Primer Name	Sequence (5′ — 3′)
**Genetic modification**	
P_{XVE}-Avr-Pita-F	TCGACCTGCAGATGCGCTATTCCCAATGTTCA
P_{XVE}-Avr-Pita-R	CATGCCTGCAGTTAACAATATTTATAACGTGC
P_{ubi}-Avr-Pita-F	TCGACCTGCAGATGGAACGCTATTCCCAATGTTCA
P_{ubi}-Avr-Pita-R	TCAGGATCCTTAACAATATTTATAACGTGC
P_{ubi}-OsCOX11-F	ACTTGGATCCATGCCGCCGCCGCCGCCGCCTTCGTT
P_{ubi}-OsCOX11-R	TCAGGATCCTTAACTGTCGTTCACCTTAAAGA
OsCOX11-U6b	TCAGCAACGTCAGCATTAAACAACACAAGCGGCAGC
OsCOX11-gRNA	TTTAATGCTGACGTTGCTGAGTTTTAGAGCTAGAAAT
OsCOX11-Ri-1F	TTACGGTACCATGGATGCTCAGCGAACTAGTC
OsCOX11-Ri-1R	CATGGTACCGTACACTCACTGCCTTAAAG
OsCOX11-Ri-2F	CATGGTACCGCTGAGGGTAAATTTCTAGT
OsCOX11-Ri-2R	TTGCGGATCCTCAGCTGAGACATCACT
**Genotyping**	
OsCOX11-In2Ex4-F	TGGAATAGCCATACAGCC
OsCOX11-In2Ex4-R	CCTTCATAGGAGCTACGTTATATGTG
OsCOX11-seq-F	GCAAATTTGCTGTAATCATGGGCTAA
**qPCR**	
Avr-Pita-qF	CCTCCTTTCTTCAACAACCC
Avr-Pita-qR	CCATCCCATTCGTAACCA
OsCOX11-qF	CAACACAGAGAGAAGTGAAGGT
OsCOX11-qR	GTGGATACACCAGTTATTGGAG
OsPAL1-qF	CCTGCCAATCTGCTGAACTA
OsPAL1-qR	TTTGAAACCTGCCACTCGTA
OsPBZ1-qF	CCGAATACGCCTAAGATGAA
OsPBZ1-qR	TCTCACGGACTCAAACGC
OsPR10-qF	AGGACTACCTCGTCGCTCA
OsPR10-qR	TTGGATTTGTCGTGGCTC
OsActin-qF	GCATCTCTCAGCACATTCCA
OsActin-qR	ACCACAGGTAGCAATAGGTA
OsUbi-qF	TTCTGGTCCTTCCACTTTCAG

OsUbi-qR	ACGATTGATTTAACCAGTCCATGA
MoPot2-qF	ACGACCCGTCTTTACTTATTTGG
MoPot2-qR	AAGTAGCGTTGGTTTTGTTGGAT
**Protein subcellular localization**	
Avr-Pita-YFP-F	TACTAAGCTTATGGAACGCTATTCCCAATG
Avr-Pita-YFP-R	TCAGGATCCCACAATATTTATAACGTGC
OsCOX11-mCh-F	CATGAAGCTTATGCCGCCGCCGCCGCCGC
OsCOX11-mCh-R	TCAGGATCCCACTGTCGTTCACCTTAAAGA
**Yeast two-hybrid**	
BD-Avr-Pita-F	TACCATATGGAACGCTATTCCCAATGTTCAGA
BD-Avr-Pita-R	TCAGGATCCTTAACAATATTTATAACGTGC
AD-OsCOX11-F	ACTTCATATGATGCCGCCGCCGCCGCCGC
AD-OsCOX11-R	TCAGGATCCTTAACTGTCGTTCACCTTAAAGA
AD-AtCOX11-F	GTACTGGAATTCATGTTAGATAGTGCCCATCGCC
AD-AtCOX11-R	CTGAGTCTCGAGTTAATTGGTTTCTTGAACTGGA
AD-ScCOX11-F	CGACGGATCCGTATGATAAGAATATGTCCCATTGTTAG
AD-SsCOX11-R	CTATGGATCCTTAATTTGAGTTGTCTTTCCTTGTGTC
AD-MoCOX11-F	ACGTTGGAATTCATGAACTCAGCAACGACGAAGC
AD-MoCOX11-R	GGATCTCTCGAGCTATGAGCTCTTCTGCTCCACC
AD-OsCOX11$^{1-198}$-F	ATGTTTTTGCTTTGAGGATCCATCGAGCTCGAGCTGCAGAT
AD-OsCOX11$^{1-198}$-R	TCGAGCTCGATGGATCCTCAAAGCAAAAACATTGTATCTTA
AD-OsCOX11$^{80-220}$-F	CATCGATCCTGAGTTTGGATCCATCGAGCTCGAGCTGCAGA
AD-OsCOX11$^{80-220}$-R	CGAGCTCGATGGATCCAAACTCAGGATCGATGTAGAAGAAC
AD-OsCOX11$^{110-220}$-F	AGATTACGCTCATATGGAGGAGAAGATCTCACGACATGCTC
AD-OsCOX11$^{110-220}$-R	GTGAGATCTTCTCCTCCATATGAGCGTAATCTGGTACGTCG
AD-OsCOX11$^{140-244}$-F	AGATTACGCTCATATGCCGTGGAAATTCATTCCAACACAGA
AD-OsCOX11$^{140-244}$-R	GAATGAATTTCCACGGCATATGAGCGTAATCTGGTACGTCG
**Pull-down**	
GST-Avr-Pita-F	CTGGTTCCGCGTGGATCCCCAGGAGAACGCTATTCCCAATGTTCA
GST-Avr-Pita-R	TCACGATGCGGCCGCTCGAGTCGATTAACAATATTTATAACGTGC
His-OsCOX11-F	ATGGCTGATATCGGATCCGAATTCCCGCCGCCGCCGCCGC
His-OsCOX11-R	TCGAGTGCGGCCGCAAGCTTGTCGTTAACTGTCGTTCACCTTAAAGA
